# Supplementary figures and images for: Identification and classification of oil and gas pipeline intru-sion events based on 1-D CNN network
Source: PLoS One. 2025 Dec 23;20(12):e0338205. doi: 10.1371/journal.pone.0338205 (PMC12725682; doi:10.1371/journal.pone.0338205)

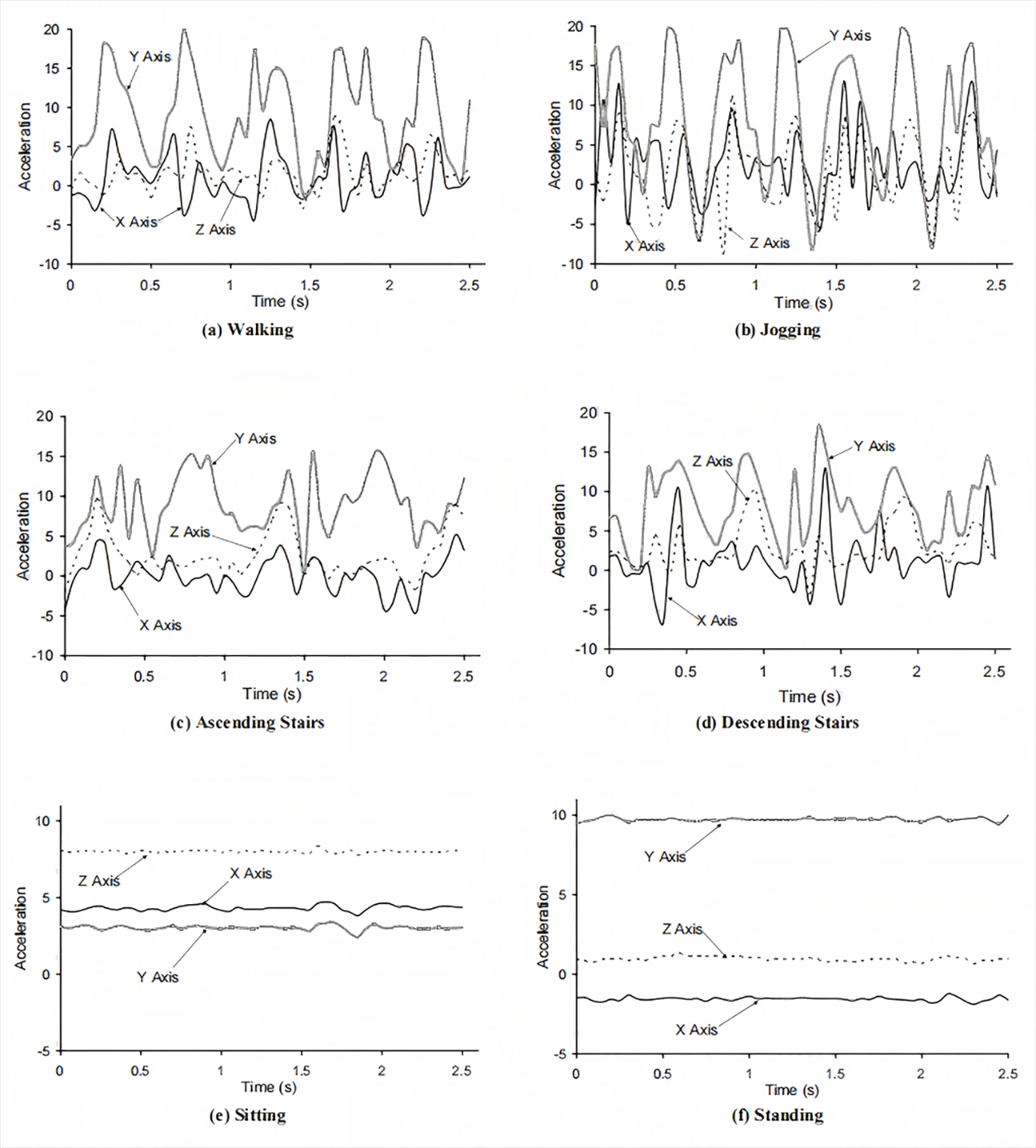

Supplement: S1 Appendix — (TIF) [file pone.0338205.s001.tif]
