# Supplementary material for: Identification and classification of oil and gas pipeline intru-sion events based on 1-D CNN network
Source: PLoS One. 2025 Dec 23;20(12):e0338205. doi: 10.1371/journal.pone.0338205 (PMC12725682; doi:10.1371/journal.pone.0338205)
Supplement: Attachment — Submitted filename: Point-by-Point Response to Reviewers (PONE-D-25-34217).pdf [file pone.0338205.s002.pdf]

Point-by-Point Response to Reviewers

Manuscript ID: PONE-D-25-41494

Original Submission: PONE-D-25-34217

Comment 1: PLOS ONE requires that all data underlying the findings be made publicly available. While we understand that your proprietary dataset cannot be shared due to confidentiality agreements, please consider the following alternatives: Provide a representative anonymized subset of the data. Release a simulated dataset that mimics the structure and characteristics of the original data. Share the code used for preprocessing, training, and evaluation of the model (e.g., via GitHub or a public repository). Without a viable mechanism for data or reproducibility support, the manuscript does not meet PLOS ONE's data sharing policy.

Actions Taken:

1.Uploaded simulated dataset to mendeley

(DOI: [10.17632 / w7nzxs593c. 1]).

2.open to all the data data network website (https://data.mendeley.com/datasets/w7nzxs593c/1)

Location:

- Section " 3.1 Data Sources" (Pages 8-9)

Comment 2: Please expand the methodological section to include: Detailed model architecture (layer parameters, kernel sizes, dropout rates, activation functions). Description of the training-validation-test splitting procedure. Strategies used to prevent overfitting (e.g., regularization, early stopping). Hyperparameter tuning procedure and optimizer settings. Number of samples per class in the proprietary dataset to assess balance.

Actions Taken:

1.there is the issue of the extended methodology section. In the method introduction part of the text, a detailed model architecture has been supplemented and explained, as well as the description process of the training-validation-testing splitting process. Added Table 1 with full layer-wise parameters .

Location:

- Section " 2.2 One-Dimensional Convolutional Neural Netword (1-D CNN)" (Page 7)

Comment 3: To substantiate the model's performance: Include statistical comparisons (e.g., confidence intervals, t-tests) between your model and baseline methods. Consider adding ablation studies to isolate the impact of handcrafted features, raw data, and transfer learning.

Actions Taken:

1. We conducted rigorous statistical analyses comparing our 1D-CNN against three classical approaches:

Model	Accuracy (Mean ± 95% CI)	F1-score	p-value (vs. 1D-CNN)
**1D-CNN (Ours)**	95.3% ± 1.1%	0.941	-
SVM (RBF Kernel)	82.7% ± 2.3%	0.812	<0.001 (Wilcoxon)
Random Forest	85.1% ± 1.9%	0.834	0.003
Logistic Regression	76.2% ± 3.5%	0.752	<0.001

Our model showed statistically significant improvement ($p<0.01$) over all baselines. The narrow confidence interval (±1.1%) demonstrates result robustness.

Detailed results in Manuscript: TABLE IV (Page 19)

2. Added Wilcoxon tests vs. SVM/RF ($p<0.01$, Table 3).

Configuration	Accuracy (±std)	Δ vs Full Model
**Full Model**	**0.953 (±0.012)**	
**Raw Signal Only**	**0.872 (±0.018)**	**-8.1%**
**Handcrafted Features Only**	**0.815 (±0.021)**	**-13.8%**
**Without Transfer Learning**	**0.901 (±0.015)**	**-5.2%**

Transfer learning improves accuracy by 5.2%, demonstrating its value for small datasets. The combined approach achieves synergistic performance beyond individual components.

Location:

- Section "3.3 Three-Class Classification Test on Oil and Gas Pipeline Data with Transfer Learning Incorporated" (Page 18)

- Supplementary TABLE Ⅲ

3.Statistical Interpretation

Effect Size Calculation: Cohen's *d* = 1.24 (Full Model vs. Features Only) → Large effect; Power analysis ($\alpha=0.05$, $\beta=0.8$) confirmed adequate sample size.

Bootstrap Validation: Resampled test set 1,000 times → 95% CI for accuracy delta: [-14.3%, -13.2%] (Features Only)

Theoretical Justification: The performance drop when removing handcrafted features (-13.8%) aligns with prior work on multimodal sensor fusion (Zhou et al., 2022, DOI:10.1016/j.patcog.2022.108985), confirming that engineered features complement learned representations. The 5.2% TL benefit reflects known cross-domain adaptation effects in DAS (Yang et al., 2023, DOI:10.1364/OE.476543).

Changes Summary:
All modifications are highlighted in the Track Changes version of the manuscript. We appreciate the opportunity to improve this work.

Sincerely,

Xiaoli Huang
Xihua University
lylihuang@xhu.edu.cn
