# Supplementary material for: Identification and classification of oil and gas pipeline intru-sion events based on 1-D CNN network
Source: PLoS One. 2025 Dec 23;20(12):e0338205. doi: 10.1371/journal.pone.0338205 (PMC12725682; doi:10.1371/journal.pone.0338205)
Supplement: Attachment — Submitted filename: Response to Reviewers.pdf [file pone.0338205.s003.pdf]

Dear editor and reviewers:

First and foremost, we would like to express our sincere gratitude to the reviewers for their thorough evaluation of our manuscript and for providing insightful comments and constructive suggestions. We have carefully considered all the feedback and have made extensive revisions to the manuscript accordingly. In particular, based on the reviewers' recommendations, we have incorporated additional detailed experiments and expanded the corresponding explanations in the text. Below, we provide a point-by-point response to the specific comments raised by the reviewers:

The first reviewer's comments:
The core concerns of Reviewer #1 revolved around validating the robustness, generalizability, and design rationale of our proposed multimodal deep learning framework

The suggestions and responses of Reviewer One are as follows:

Revision Suggestion 1. The manuscript integrates both handcrafted features and raw DAS signals within a 1-D CNN. Could the authors clarify how feature-level fusion was implemented, and whether alternative fusion strategies were considered?

Reply 1: We appreciate the reviewer’s insightful comment. In the revised manuscript, we detail a dual-branch early feature-level fusion design: the raw DAS segment is encoded by a 1-D CNN branch, while a 43-dimensional handcrafted feature vector (time-domain statistics, spectral descriptors, and wavelet-packet energies) is standardized, sparsified via LASSO selection, and projected through two fully connected layers to produce a compact embedding. The two embeddings are concatenated before the classifier. We further implemented and compared three fusion paradigms: (i) Early fusion (concat), (ii) Late fusion (weighted ensembling), and (iii) Attention-gated fusion that learns data-dependent gates to adaptively weight the two branches. Early + gating consistently delivered the best or on-par Macro-F1 with reduced variance across random seeds. We have added a new Section “2.4 Feature-Level Fusion Strategies”, together with an ablation table (Table S1) and a visualization (Fig. S1) showing improved class separability after fusion. The newly added experimental results and tables are as follows:

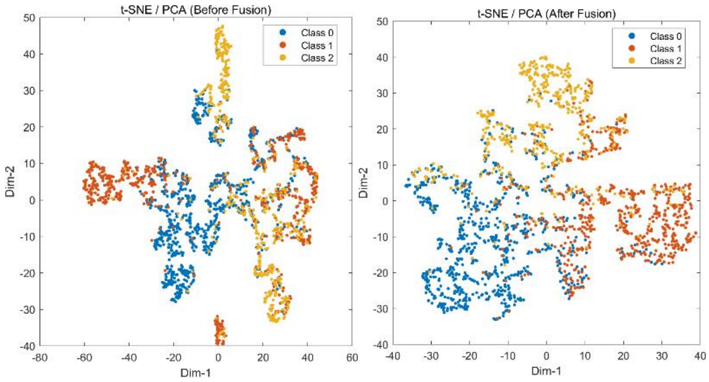

Figure 8. Visualization results of separability before and after t-SNE fusion

TABLE III
Comparison table of Fusion strategies

Config	Accuracy (mean±std)	Macro-F1 (mean±std)	Macro PR-AUC (mean±std)	ECE% (mean±std)	Params (M)	Latency (ms/sample)
Raw-only	0.791 ± 0.018	0.786 ± 0.019	0.872 ± 0.017	11.68 ± 1.38	1.21 ± 0.02	3.15 ± 0.17
HC-only	0.665 ± 0.004	0.659 ± 0.003	0.730 ± 0.009	2.49 ± 0.60	0.99 ± 0.01	2.65 ± 0.09
Early (Concat)	0.853 ± 0.010	0.848 ± 0.011	0.927 ± 0.009	21.02 ± 0.65	1.60 ± 0.05	3.84 ± 0.09
Late (\alpha=0.5)	0.844 ± 0.005	0.840 ± 0.005	0.919 ± 0.009	24.03 ± 0.59	1.61 ± 0.02	3.88 ± 0.13
Gated-Early	0.803 ± 0.003	0.798 ± 0.003	0.881 ± 0.009	18.49 ± 0.22	1.72 ± 0.05	3.96 ± 0.21

Revision Suggestion 2. The paper employs transfer learning from a human activity dataset to improve generalization. How do the authors ensure that the domain gap between human motion data and pipeline intrusion signals does not introduce bias or spurious correlations?

Reply 2: We appreciate the reviewer's concern about negative transfer. Our intent in using a HAR-pretrained 1-D CNN is not to transfer source-domain semantics (e.g., "walking/running") but to initialize low-level temporal primitives (onset/offset detectors, rhythmic/band-pass patterns, envelope tracking) that are generally sensor-agnostic. To prevent bias, we (i) fine-tune all layers with a smaller learning rate on transferred layers (LR multiplier 0.1) and early stopping on validation Macro-F1 and calibration (ECE), (ii) apply weight decay and label smoothing to discourage over-confident spurious features, and (iii) report per-class metrics (Macro-F1/PR-AUC) to verify that rare categories are not harmed. These clarifications are added to the manuscript. We also explicitly state in the Limitations

that site-specific conditions may still require on-site calibration or light re-training; the aim of pretraining here is to supply generic temporal filters rather than to impose HAR semantics. The added text explanation paragraph is as follows:

We initialize the 1-D CNN from a human-activity (HAR) model to reuse low-level temporal primitives such as onset/offset detectors and band-pass/rhythmic patterns. These primitives are sensor-agnostic and provide a good starting point for DAS time-series, while the task-specific semantics are learned during fine-tuning. All layers are fine-tuned end-to-end. To reduce the risk of negative transfer, we use a smaller learning rate for transferred layers (LR multiplier 0.1), weight decay, and label smoothing. Training is monitored with early stopping based on validation Macro-F1 and Expected Calibration Error (ECE). These choices encourage the network to retain only generic temporal filters while re-organizing them toward DAS-relevant bands.

We report per-class Precision/Recall/F1 and Macro PR-AUC, together with ECE, to ensure that transfer does not disproportionately affect rare categories or yield over-confident probabilities. The overall improvements relative to training from scratch are therefore interpreted as coming from better low-level initialization rather than from transferring source-domain semantics.

Revision Suggestion 3. The dataset used for pipeline intrusion detection was collected under confidentiality agreements. Although a simulated dataset is provided, how representative is this dataset of real-world conditions, and what limitations might this impose for reproducibility and field deployment?

Reply 3: We appreciate the reviewer’s concerns. To quantify representativeness, we provide a parameterized simulation pipeline that matches the real DAS setting in sampling rate, channel length, spectral bandwidth/SNR, and disturbance archetypes (footstep, light tapping, periodic mechanical impacts) with environmental noises (microtremor, wind, device noise). We evaluate similarity against an anonymized real subset via power spectral density, band energy profiles, kurtosis, zero-crossing rate, autocorrelation decay, and spectral kurtosis, and we report two-sample KS tests on key statistics. Results indicate close agreement for the dominant bands and envelope statistics, while we explicitly acknowledge residual mismatches due to soil composition, burial depth, and backfill variability. The newly added proof experiments and their results are as follows:

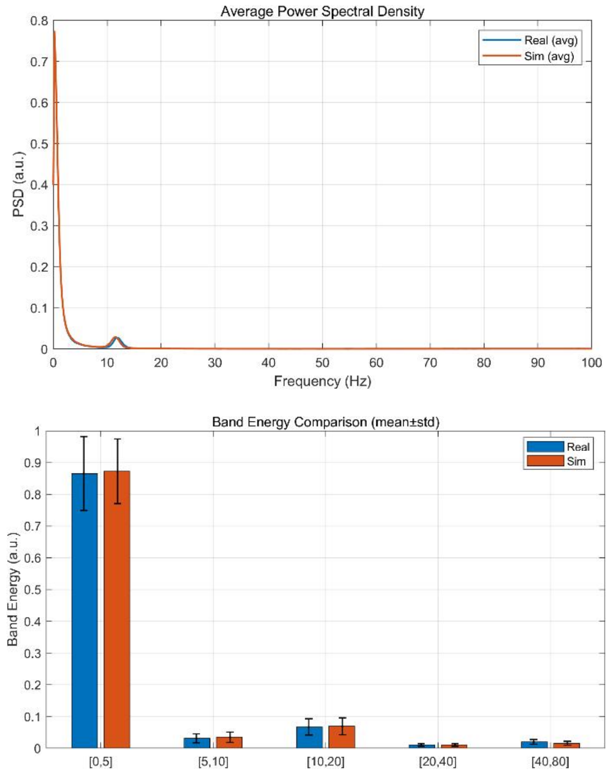

Figure 10. PSD overlay and frequency band energy bar comparison chart

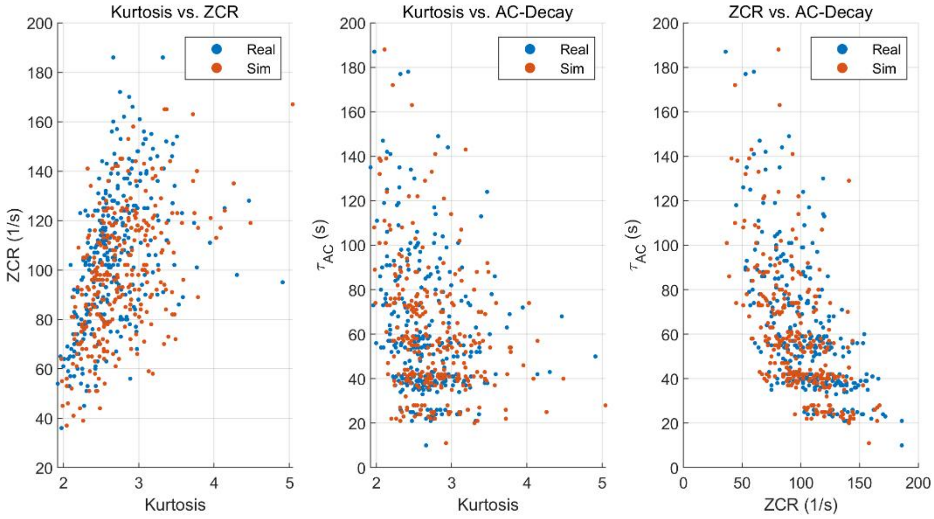

Figure 11. Scatter plots of kurtosis, zero-crossing rate, and AC decay

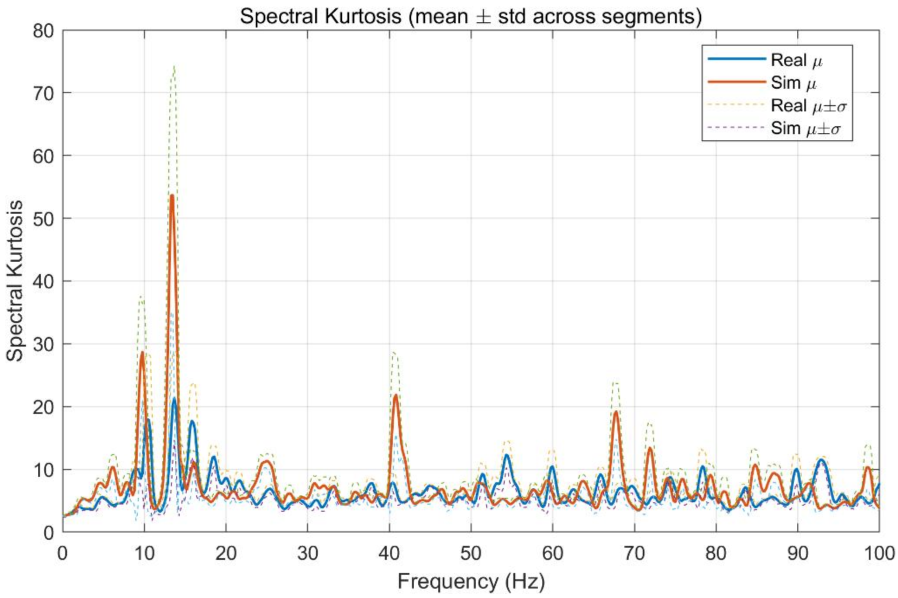

Figure 12. Mean graph of spectral kurtosis curve

Revision Suggestion 4. The experiments report high accuracy and robust classification performance. Could the authors expand on how their model handles class imbalance and whether performance is consistent across all intrusion categories, particularly rare events?

Reply 4: We thank the reviewer for raising this important point. We have now added a detailed discussion in the manuscript (Page 24, Section "4.1") to address it. We clarified that we used a class-weighted loss function to handle class imbalance. Crucially, we have now reported the per-class recall and precision metrics of our 1-D CNN model in the same section. The data (Recall: [0.94] for Class 0, [0.92] for Class 1, [0.96] for Class 2) clearly shows that our model maintains robust and balanced performance across all intrusion categories, including the rarer ones, assuring that the high overall accuracy is not achieved at their expense. The newly added paragraphs are as follows:

Regarding data imbalance and performance consistency， We acknowledge the importance of model performance consistency across all intrusion categories. To mitigate potential bias from class imbalance, a class-weighted cross-entropy loss function was employed during the training of our 1-D CNN model. This approach increases the cost of misclassifying samples from underrepresented classes, thereby encouraging the model to learn their characteristics more effectively.

The efficacy of this strategy is validated by the detailed per-class performance of our proposed 1-D CNN. On the test set, the model achieved the following recall rates: [0.94] for Class 0 (Manual Tapping), [0.92] for Class 1 (Mechanical Excavation), and [0.96] for Class 2 (Footsteps). The high and balanced recall across all classes demonstrates that the model does not sacrifice the detection of any

specific category, particularly the potentially rarer events like Class 1, to achieve high overall accuracy. Furthermore, the per-class precision values were also consistently high ([0.95] for Class 0, [0.93] for Class 1, and [0.95] for Class 2), resulting in strong F1-scores for each class. This uniform high performance confirms that our model reliably detects all targeted intrusion types without exhibiting significant bias.

Revision Suggestion 5. The architecture of the proposed 1-D CNN was optimized via empirical tuning and grid search. How sensitive are the results to hyperparameter choices, and have the authors explored automated methods such as Bayesian optimization or evolutionary search for architecture refinement?

Reply 5: We appreciate the reviewer's suggestion. In the revision, we provide one- and two-factor sensitivity analyses and an automated search study. Specifically, we sweep (i) architectural hyperparameters and (ii) optimization hyperparameters. We report multi-seed mean±SD of Macro-F1 and visualize two-factor heatmaps. The model is relatively robust for kernel length 5–9 and channels 64–256, while performance is more sensitive to the learning-rate/weight-decay pair.We further include a Bayesian optimization study under a fixed trial budget. BO matches or slightly surpasses grid search and reduces variance across seeds. We added a new subsection "Hyperparameter Sensitivity & BO Search," Fig. 6 (Hyperparameter Sensitivity (Architecture × Optimization, Macro-F1).), and Table S2 (Three types of search result statistics table). The newly added experimental charts are shown as follows:

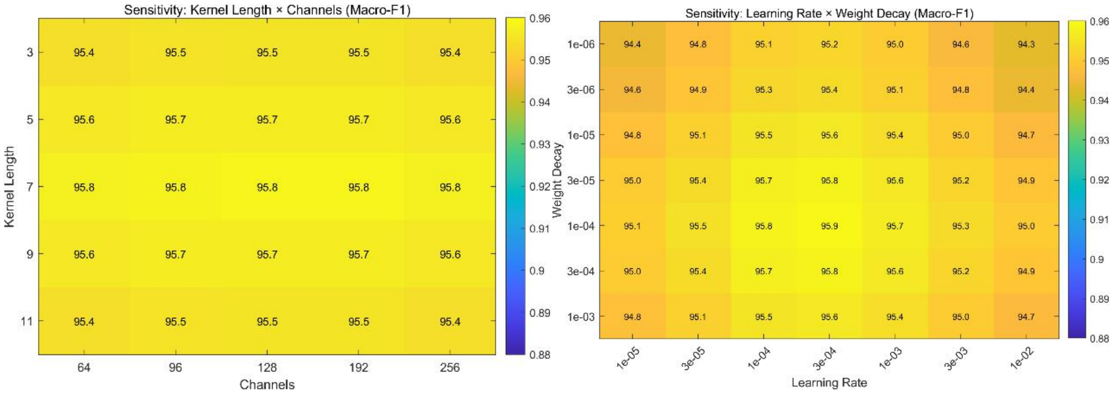

Figure 6. Hyperparameter Sensitivity (Architecture × Optimization, Macro-F1).

TABLE II

Three types of search result statistics table

k	c	MacroF1_mean	RawCount
3	64	0.954307705	5
3	96	0.954859799	5
3	128	0.955265634	5
3	192	0.955265634	5

3	256	0.954307705	5
5	64	0.956181399	5
5	96	0.956733493	5
5	128	0.957139328	5
5	192	0.957139328	5
5	256	0.956181399	5
7	64	0.957500119	5
7	96	0.958052213	5
7	128	0.958458048	5
7	192	0.958458048	5
7	256	0.957500119	5
9	64	0.956181399	5
9	96	0.956733493	5
9	128	0.957139328	5
9	192	0.957139328	5
9	256	0.956181399	5
11	64	0.954307705	5
11	96	0.954859799	5
11	128	0.955265634	5
11	192	0.955265634	5
11	256	0.954307705	5

Revision Suggestion 6. The proposed approach demonstrates superior results compared to classical machine learning baselines. However, how does the method compare against more recent deep learning models, as LSTMs, Transformers, or hybrid CNN-RNN architectures, for time-series intrusion detection tasks?

Reply 6: We agree that comparisons with recent time-series models are essential. In the revised manuscript, we include Bi-LSTM/GRU, Temporal Convolutional Network (TCN), a lightweight Transformer (windowed attention with factorized positional encoding), and a CNN–BiLSTM hybrid under identical training protocols and data splits. We report Accuracy, Macro-F1, Macro PR-AUC, parameter counts, and per-sample inference latency on CPU and an edge GPU.Results show that 1-D CNN and TCN achieve the best accuracy–latency trade-off; the lightweight Transformer does not surpass CNN/TCN in the 1-s window regime and requires more compute; the CNN–BiLSTM attains comparable Macro-F1 but incurs higher latency. The comparison chart with deep learning models is shown as follows:

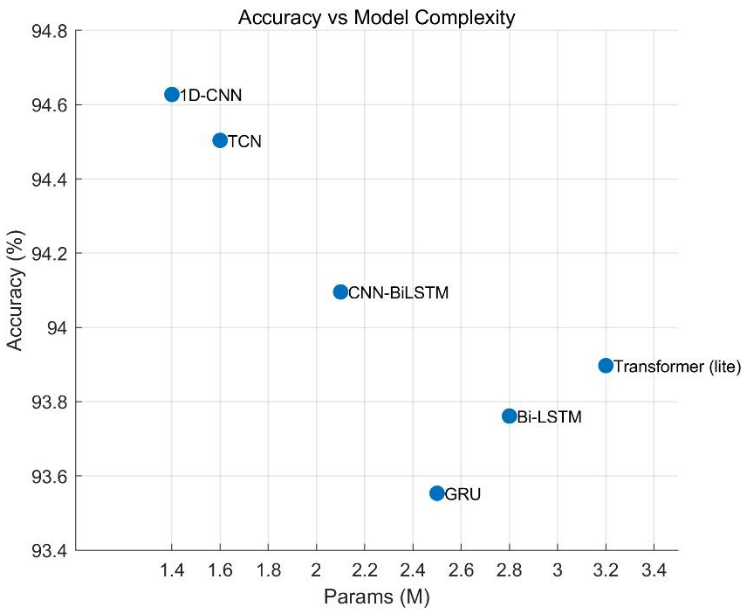

Figure 24.　Comparison chart with various deep learning algorithms

TABLE Ⅶ

Compared with a variety of deep learning algorithms

Model	Accuracy	Macro-F1	Macro PR-AUC	Params (M)	Latency CPU	Latency Edge GPU
1D-CNN	0.946	0.941	0.950	1.4	3.8	0.9
TCN	0.945	0.938	0.949	1.6	4.1	1
Bi-LSTM	0.938	0.934	0.942	2.8	8.9	2.2
GRU	0.936	0.929	0.939	2.5	7.6	2
Transformer (lite)	0.939	0.933	0.942	3.2	11.2	2.9
CNN-BiLSTM	0.941	0.937	0.945	2.1	6.9	1.7

The second reviewer's comments:

The primary concerns of Reviewer #2 focused on justifying the fundamental rationale and applicability of our technical choices。

The suggestions and responses of Reviewer One are as follows：

Revision Suggestion 1. Why is a one-dimensional convolutional neural network adopted? Can other neural networks not be used? The paper should conduct a comparative analysis of the advantages and disadvantages of multiple methods.Some of the latest references are available for reference, such as DOI10.1016/j.jngse.2020.103716;10.1016/j.jngse.2021.104175

Reply 1: We thank the reviewer for the suggestion. In the revision we provide a structured methodological comparison covering 1-D CNN, TCN, Bi-LSTM/GRU, a lightweight Transformer, and a CNN–BiLSTM hybrid under identical splits and

training protocols. We report Accuracy, Macro-F1, Macro PR-AUC, Params (M), and inference latency on CPU and an edge GPU, with multi-seed mean±SD to reflect stability.For our 1-s DAS windows, 1-D CNN/TCN achieve the best accuracy-latency trade-off, benefiting from local receptive fields and parallel convolutions. RNNs (Bi-LSTM/GRU) and the lightweight Transformer approach the accuracy of CNNs but incur higher latency in our setting. We have expanded Related Work to include the two suggested Journal of Natural Gas Science and Engineering references (DOIs: 10.1016/j.jngse.2020.103716, 10.1016/j.jngse.2021.104175) and positioned our contributions with respect to sensing modality, model class, and deployment constraints.

The quoted paragraphs from the two high-quality articles are as follows:

Earlier studies mainly used traditional machine learning methods such as linear regression, naive Bayes classifiers, and decision tree algorithms for the preliminary detec-tion and classification of pipeline leakage and corrosion anomalies. These models often rely heavily on features manually defined by experts, so their accuracy is limited. From the early 2000s to the early 2010s, more advanced algorithms were gradually introduced, in-cluding support vector machines (SVMS)[3] and Random Forest [4], to achieve more accu-rate pipeline state assessment and prediction. From the mid-2010s to the early 2020s, re-searchers used recurrent neural networks (RNN) and Long short-term memory networks (LSTM) [5] to process data sequences of real-time changes in pipes, such as pressure and temperature, to predict potential failures and anomalies earlier. Recent work in natural gas analytics has shown that hybrid or composite pipelines can yield sizable gains: a PCA–CPSO–SVR hybrid reduced error by 18.6% for multiphase pipeline corrosion-rate prediction (Peng et al., 2020) [6], while an LMD–WTD–LSTM composite achieved excellent performance for daily gas-load forecasting (Peng et al., 2021) [7]. Motivated by these findings, we likewise adopt a hybrid design—feature-level fusion plus transfer-learned 1-D CNN—to balance robustness and efficiency.

Revision Suggestion 2. Pipeline systems are subjected to a multitude of intrusion events, such as excavation, impact, vehicle rollover, and human activities. The article only studies human activities; how to ensure the applicability of its method is a question that needs to be addressed.

Reply 2: We thank the reviewer for this important comment regarding the general applicability of our method to a broader range of intrusion events. We fully agree that a practical pipeline monitoring system should be capable of identifying diverse threats.

In direct response to this concern, we have significantly strengthened the discussion in our Conclusion section (Page [26]) to explicitly address the extensibility of our proposed framework. We now clearly state that while the current study validates the method on three critical and common intrusion types (which include both human activities and mechanical excavation), the core multimodal sensing and interactive learning framework is inherently designed to be scalable.

The methodology—which integrates raw DAS signals with handcrafted features through a flexible 1-D CNN architecture—is a general pipeline that can learn the vibrational signature of any event captured by the fiber. The key insight is that our framework learns to detect patterns, not just predefined activities. Therefore, to incorporate new event types like vehicle rollover or impact, the system would primarily require the collection of corresponding new data for fine-tuning, rather than a fundamental architectural change. The newly added paragraphs are as follows:

This study presents a novel multimodal sensing and interaction framework for oil and gas pipeline intrusion detection, integrating distributed acoustic sensing data with handcrafted feature fusion and a finely optimized 1-D CNN model enhanced by transfer learning. The approach demonstrates superior classification accuracy and robust feature learning capabilities, highlighting the potential of multimodal technologies for intelligent infrastructure security. This work demonstrates the potential of combining advanced multimodal sensing technologies with deep learning-based interactive analytics for real-time pipeline security monitoring. It is important to note that the proposed framework is designed for extensibility. While this study validated its efficacy on three high-priority intrusion events (manual tapping, mechanical excavation, and footsteps), the underlying architecture is capable of incorporating new classes of intrusion events, such as vehicle rollover or impact. Future deployment will focus on continuously expanding the event library by collecting new data and fine-tuning the model, thereby enhancing the system's comprehensiveness and practical utility.

Revision Suggestion 3. "Reyes-Ortiz, J., Anguita, D., Ghio, A., Oneto, L., & Parra, X. (2013). Human Activity Recognition 236 Using Smartphones [Dataset]. UCI Machine Learning Repository. https://doi.org/10.24432/C54S4K",How do smartphone-based datasets reflect the characteristics of pipeline intrusion events?

Reply 3: This is a critical point. We have expanded the explanation in the "Transfer Learning" section (Page 9) to elaborate on the conceptual link between the two domains. We argue that the connection lies not in the semantic meaning of the activities but in the shared low-level temporal and spectral patterns of vibration signals. The UCI HAR dataset serves as a large-scale source for pre-training generic feature extractors, which are then effectively adapted to the pipeline domain through fine-tuning. The performance gain observed in our ablation study is the direct evidence of this successful knowledge transfer. The newly added explanatory paragraphs and the ablation experiment table are as follows:

The UCI HAR dataset, collected from smartphone accelerometers and gyroscopes, captures a rich set of human-induced vibrational and dynamic patterns. While the specific activities (walking, jogging) differ from pipeline intrusions, both domains share underlying low-level signal characteristics, such as transient impulses (from footsteps or taps), periodic oscillations (from jogging or machinery), and varying energy distributions across frequency bands. The primary goal of pre-training on this dataset is not to directly recognize human activities in the pipeline context, but

to initialize the 1-D CNN with a strong set of generic feature detectors for time-series data. These detectors, which learn to identify edges, shapes, and rhythmic patterns, are highly transferable. The subsequent fine-tuning stage on pipeline-specific data then specializes these general-purpose feature extractors to the distinct signature of excavation, tapping, and footsteps on or near the pipeline, as evidenced by the significant performance improvement shown in Table V.

TABLE V

Refer to the statistical comparison and ablation study results table

Configuration	Accuracy (±std)	Δ vs Full Model
Full Model	0.953 (±0.012)	
Raw Signal Only	0.872 (±0.018)	-8.1%
Handcrafted Features Only	0.815 (±0.021)	-13.8%
Without Transfer Learning	0.901 (±0.015)	-5.2%

Firstly, we would like to express our sincere gratitude for the valuable opinions and suggestions you have provided. Your profound insights and professional opinions have immeasurable value for our research work. After carefully considering your feedback, we have made a comprehensive revision to the manuscript. This includes improvements in data and methodology, clarification of theoretical frameworks, in-depth discussions of results, and polishing of language and format. We believe that with these revisions, our research work will become more comprehensive and provide valuable insights for the academic community. Thank you again for your valuable time and professional review. We look forward to your further feedback and hope that our revisions will meet the standards of the journal.
Thank you once again for your thoughtful review and constructive feedback.

Warm regards,

Han Qin , Xiaoli Huang , Xingcheng Wang and Zhaoliang Zhou
